# Time to stand up faster: underutilization of real-world sit-to-stand transition velocity in aging research

**DOI:** 10.3389/fragi.2026.1639286

**Published:** 2026-01-22

**Authors:** Myles W. O’Brien

**Affiliations:** 1 Centre de formaiton médicale du Nouveau-Brunswick, Université de Moncton and Université de Sherbrooke, Moncton, NB, Canada; 2 Department of Medicine, Université de Sherbrooke, Sherbrooke, QC, Canada

**Keywords:** accelerometry, physical frailty, physical function, posture, standing

## Introduction

Frailty reflects a decrease in physiological reserve, increasing vulnerability to adverse events following minor illnesses to health insults (Mitnitski et al., 2001). Higher levels of frailty correspond to age-related health deficits that incur a greater likelihood of physical immobility, cognitive disorders, and premature mortality (Romero-Ortuno et al., 2016). One of the most effective strategies for preventing and managing frailty is regular physical exercise. Specifically, exercise had a more consistent positive effect on frailty than diet, geriatric assessments, and optimal medication control (Negm et al., 2019). However, due to the development of more detailed movement guidelines (Rollo et al., 2022) and low population rates of engaging in exercise, there is greater interest in targeting postures that comprise most of our waking hours and specifically breaking up sedentary behaviors with upright activities. There is a need to study innovative methods to monitor and intervene in order to promote healthier movement patterns among frail populations.

**FIGURE 1:**
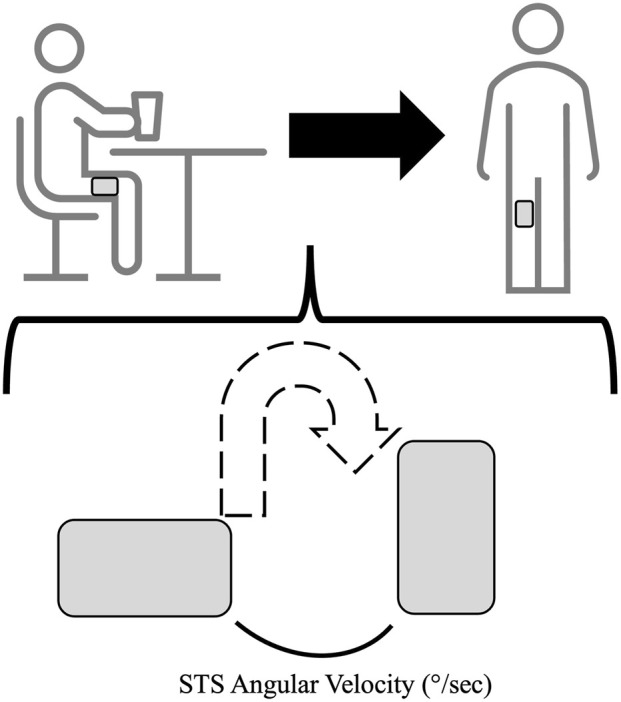
Conceptual example of demonstrating the rudimentary nature of accelerometry-derived sit-to-stand velocity.

Advances in wearable monitors have equipped researchers with relatively precise tools to measure people’s activity and postures to fractions of a second (see Figure 1). Monitors worn on the thigh have equipped researchers with the ability to compute both the number of sit-to-stand (STS) transitions and their velocity. Over the past decade, sedentary breaks, defined as the number of STS transitions, have garnered substantial attention, with studies showing that more frequent STS breaks are positively associated with several health markers (Healy et al., 2008). For example, more frequent STS breaks were associated with better waist circumference (Healy et al., 2008), triglycerides (Healy et al., 2008), and blood pressure regulation (O’Brien et al., 2021), independent of overall sedentary activity. However, the velocity at which these STS transitions occur is not often considered. With aging, the ability to stand up independently is paramount. Adverse physiological changes in the musculoskeletal system, cardiovascular system, and neurological systems occur with sedentary aging that make the STS transition slow. This opinion article outlines the potential utility of STS-velocity (STSv) in aging research, with the objective of highlighting its value and promoting further discourse on this metric and its inclusion in researchers’ repertoire of physical behavior outcomes for studies of older adults.

## Discussion: intensity complementing frequency

Older adults tend to break up their sedentary time less frequently than younger adults (O’Brien et al., 2021). The frequency of STS transitions is highly variable, and examining how often they occur may be missing an important characteristic of this behavior. Using aerobic activity as an example, the frequency of STS may be analogous to step count frequency, whereas STSv may be conceptualized as minutes of moderate-vigorous aerobic physical activity. Herein, the intensity at which stepping occurs is, at minimum, equally important to characterizing movement, and the evidence base for physical activity intensity is substantial. For example, movement guidelines for physical activity rely on intensity-related physical activity rather than frequency (e.g., step counts) (Rollo et al., 2022). Using this logic, the velocity by which each STS occurs may provide valuable information that might be more impactful than STS frequency alone. Accordingly, a faster STS transition provokes a greater cardiovascular and cerebrovascular response and requires greater muscular power (O’Connor et al., 2020). Of note, pronounced decreases in arterial pressure, decreases in prefrontal cortex oxygenation, and increases in the heart rate are observed for 20–30 s, following a single STS transition (O’Connor et al., 2020). Thresholds for what constitutes slow/fast or thresholds similar to activity (light, moderate, and vigorous velocities) are an important area for establishing this understudied metric.

The decision to stand up is inherently influenced by several factors and often not a reflective or well-thought-out choice unless the person has mobility issues. It is plausible that between-group studies (e.g., frail vs. non-frail) or within-group interventions (e.g., exercise training study in older adults) may conclude no differences/changes in postural measures based on the frequency of STS transitions. It is posited that STSv is particularly relevant for an older adult population and that the transition from chair rising being an automatic task to one that is a conscious decision is especially important for frail, older adults. If such transitions became faster but STSv was not quantified in single-group or post-intervention analysis, the null hypothesis would be accepted, leading to a type II error concluded. Considering STSv may improve researchers’ toolboxes for understanding clinically relevant questions related to aging and frailty.

## Discussion: degree of lower-limb functional capacity

Based on laboratory testing in highly controlled conditions, it is well-established that faster STSv (e.g., via a 5-time STS test) is associated with better mobility (Whitney et al., 2005) and a reduction in fall risk (Tateoka et al., 2024), or *vice versa*. This test reflects their maximal level of repeatedly doing the same action in a very short period of time, providing clinically relevant information. However, how well this reflects their usual STSv when not under the direct supervision of an evaluator is unclear. While there is some evidence to indicate that maximal STSv and habitual STSv are positively correlated (Löppönen et al., 2023; Löppönen et al., 2024), habitual STS is markedly lower, plausibly more variable, and more accurately conveys the true physical behaviors of the person. Studies of small samples have documented that older adults and those with higher physical frailty levels (via the phenotype) exhibit slower STSv (Löppönen et al., 2023; Ganea et al., 2012). Standing up faster and more frequently would likely contribute to a higher maximal STSv (or an attenuated decline over time). Relatedly, the detailed information available for each transition allows for in-depth analysis on the STS patterns throughout the day. For example, most individuals take between 10 and 90 STS transitions per day (or ∼70–630 per week) (O’Brien et al., 2021; Shivgulam et al., 2022); studying free-living STSv provides insights into changes throughout the day and from day-to-day and lends itself to the development of potential lifestyle interventions. In relation to aging and frailty, STSv may closely reflect musculoskeletal function, and identifying strategies to ensure STSv is sufficiently high could be an important measure of overall mobility and reducing the risk of disability for geriatricians and other members of the healthcare team. It is posited that STSv, or the ability to stand up quickly, may serve as a clinically relevant endpoint in studies aimed at understanding age- and frailty-related alterations in cardiovascular, neural, and muscular function

## Discussion: quantifying free-living STSv

Advancements in thigh-worn accelerometry hardware and software have equipped postural researchers with tools to measure habitual standing and sedentary time. With the inclusion of sedentary time into movement guidelines (Rollo et al., 2022), more researchers studying physical behaviors are opting for monitors worn on the thigh compared to other locations that cannot or more indirectly assess sedentary postures (i.e., waist or wrist). Importantly, these monitors are well-tolerated by older adults and can be worn 24-h/day for a full week with transparent dressing even on older, clinical populations with fragile skin (O’Brien et al., 2022). Whether a sufficient number of transitions or wear periods longer than 1 week are needed to reliably assess STSv is unclear. Methodological studies examining the practical and technical aspects of STSv are therefore needed.

Computationally, commercially available software for thigh-worn monitors provides the raw acceleration data. Currently available commercial software put forth by accelerometer companies does not quantify STSv, but it would be unsurprising for this to change with an increased inclusion of this metric among researchers. Rather, several research groups have developed their own analysis programs and engaged in open-sciences practices to enable others to measure STSv (Rayner et al., 2025; Löppönen et al., 2021; Pickford et al., 2019). Eventually, it is expected that a harmonized analytical approach will be adopted (e.g., similar filtering, sampling rate requirements, and strategies to denote the beginning and end of an STS transition). Currently, few studies have been conducted examining free-living STSv (Rayner et al., 2025), but such information can be readily determined from thigh accelerometers and would inherently complement both STS frequency and overall sedentary time. Whether there are methodological considerations that would challenge the validity of STSv measures are unclear. These could include, for example, chronic conditions or health issues that create unilateral imbalances (e.g., unilateral knee osteoarthritis). Similarly, there may be utility in studying STSv beyond simply a median or peak velocity, and the acceleration profile or STSv phenotype could hypothetically provide useful information. Details on the technical aspects of measuring STSv and the heterogeneity of the field are beyond the scope of this study and are discussed elsewhere (Rayner et al., 2025). Regardless, this potentially important metric can be determined prospectively and retrospectively. Devices positioned on the thigh for free-living conditions (e.g., activPAL, Axivity, and Fibion SENS) that provide the raw accelerations could be used to determine STSv at home or in outpatient settings. Current analysis pipelines require data to be downloaded and analyzed using custom software programs. Advancements in this analytical strategy could enable practitioners (e.g., physiotherapists, physicians, and kinesiologists) to rapidly determine a person’s STSv in-person or virtually, which could help guide care treatment planning or movement recommendations. Subsequent guidelines or recommendations to help providers interpret such data would be practically useful. As devices become more advanced and progressively easier to implement in clinical settings, the utility of this metric could provide unique information on patients’ mobility. Once a greater awareness of STSv is achieved, sufficient understanding of its validity/reliability and measurement characteristics are optimized, and there is great potential for answering research questions on the aging process and testing models to improve physical function and reduce fall risk among geriatric populations.

## Conclusion

The ability to stand up quickly is a fundamental aspect of human movement that requires coordinated physiological effort. Preliminary evidence indicates that the velocity of the STS transition may be impaired with aging and higher levels of frailty. It is argued that the intensity at which STS transition occur may be as important as, if not more than, the frequency of transitions. Greater integration of STSv, along with further investigation of its clinical and methodological aspects, is needed to inform clinical practice and advance scientific research.
